# When can we measure stress noninvasively? Postdeposition effects on a fecal stress metric confound a multiregional assessment

**DOI:** 10.1002/ece3.1857

**Published:** 2016-01-09

**Authors:** Jennifer L. Wilkening, Chris Ray, Johanna Varner

**Affiliations:** ^1^ Department of Ecology and Evolutionary BiologyUniversity of ColoradoBoulderColorado; ^2^ Institute of Arctic and Alpine ResearchUniversity of ColoradoBoulderColorado; ^3^ Department of BiologyUniversity of UtahSalt Lake CityUtah

**Keywords:** Climate sensitive mammal, localized environmental effects, microclimate, multiregional assessment, noninvasive sampling, physiological stress

## Abstract

Measurement of stress hormone metabolites in fecal samples has become a common method to assess physiological stress in wildlife populations. Glucocorticoid metabolite (GCM) measurements can be collected noninvasively, and studies relating this stress metric to anthropogenic disturbance are increasing. However, environmental characteristics (e.g., temperature) can alter measured GCM concentration when fecal samples cannot be collected immediately after defecation. This effect can confound efforts to separate environmental factors causing predeposition physiological stress in an individual from those acting on a fecal sample postdeposition. We used fecal samples from American pikas (*Ochotona princeps*) to examine the influence of environmental conditions on GCM concentration by (1) comparing GCM concentration measured in freshly collected control samples to those placed in natural habitats for timed exposure, and (2) relating GCM concentration in samples collected noninvasively throughout the western United States to local environmental characteristics measured before and after deposition. Our timed‐exposure trials clarified the spatial scale at which exposure to environmental factors postdeposition influences GCM concentration in pika feces. Also, fecal samples collected from occupied pika habitats throughout the species' range revealed significant relationships between GCM and metrics of climate during the postdeposition period (maximum temperature, minimum temperature, and precipitation during the month of sample collection). Conversely, we found no such relationships between GCM and metrics of climate during the predeposition period (prior to the month of sample collection). Together, these results indicate that noninvasive measurement of physiological stress in pikas across the western US may be confounded by climatic conditions in the postdeposition environment when samples cannot be collected immediately after defecation. Our results reiterate the importance of considering postdeposition environmental influences on this stress metric, especially in multiregional comparisons. However, measurements of fecal GCM concentration should prove useful for population monitoring within an eco‐region or when postdeposition exposure can be minimized.

## Introduction

Much wildlife conservation research focuses on identifying impacts of anthropogenic habitat loss, fragmentation, and degradation on species health and persistence. Habitat can be defined as the set of environmental conditions (e.g., temperature, precipitation) and resources (e.g., shelter, food) that determine the presence, survival, and reproduction of a population (Block and Brennan 1993; Hall et al. 1997). Changes in habitat resulting from seasonal variation or stochastic events can cause physiological stress (Munck et al. 1984; Moberg 2000; Hik et al. 2001), and an evolved stress response is often what enables animals to successfully respond to these events (Romero 2002). Recent reviews have highlighted numerous studies on effects of human activities and related environmental disturbance on stress in vertebrates (Busch and Hayward 2009; Dantzer et al. 2014). However, for many other species, the effects of increased physiological stress resulting from anthropogenic habitat change are still unknown.

Glucocorticoid (GC) stress hormones and their metabolites can be measured in physiological samples such as blood, feces, or urine. In response to a stressor, vertebrates release glucocorticoid (cortisol or corticosterone) hormones into the blood stream. This release of GCs in response to short‐term stress permits rapid energy mobilization and behavioral change, which can result in improved survival and reproduction. However, continued release of GCs resulting from persistent chronic stress can lead to adverse health effects, immunosuppression, and reduced reproduction, which ultimately decrease fitness (Riley 1981; Munck et al. 1984; McEwen and Sapolsky 1995; Boonstra 2005).

Interest in the effects of anthropogenic disturbance on the endocrine stress response has grown, and recent studies have focused on how habitat fragmentation (Rangel‐Negrin et al. 2009), degradation (Balestri et al. 2014; Rizo‐Aguilar et al. 2014), and proximity to urban environments (French et al. 2008; Fokidis et al. 2011; Zhang et al. 2011) influence physiological stress in wildlife. Similarly, the quantification of physiological stress in some species has been used as a bio‐indicator of habitat quality. For example, pollution has been linked to elevated GCs in southern toads (*Bufo terrestris*; Hopkins et al. 1997) and Galapagos marine iguanas (*Amblyrhynchus cristatus*; Wikelski et al. 2001), and differences in forest habitat structure resulting from past logging activity have been correlated with higher GC concentration in northern spotted owls (*Strix occidentalis caurina*; Wasser et al. 1997). This use of a physiological stress measure as a bio‐monitor has the potential to improve our ability to predict which properties of habitat quality will have the greatest impact on populations. The growing field of conservation physiology integrates physiological information with ecological data to inform conservation management, and physiological stress metrics are being incorporated into conservation plans for many wildlife species (Busch and Hayward 2009; Cooke et al. 2013; Dantzer et al. 2014).

Glucocorticoids circulating in the body are typically metabolized by the liver, and then excreted into the gut as metabolites (Taylor 1971; Palme et al. 1996; Mostl and Palme 2002). Glucocorticoid metabolites (GCMs) can be detected in the feces of birds and mammals. Noninvasive sampling of fecal GCM is advantageous in several ways. First, in many species, fecal samples can be collected relatively easily without disturbing or endangering the animal, which facilitates repeated sampling over time (Mostl and Palme 2002; Millspaugh and Washburn 2004). Second, fecal samples collected noninvasively are not biased by capture induced stress, and so may provide a more precise assessment of the endocrine condition of an animal (Harper and Austad 2000; Millspaugh et al. 2001; Touma and Palme 2005). Finally, relative to GC levels in the blood, fecal GCM measurements may be less affected by the pulsatile nature of hormone secretion (Palme et al. 1996, 2005; Harper and Austad 2000).

One major caveat associated with fecal GCM measurement is that environmental characteristics can alter GCM concentration in exposed feces if samples are not frozen (or similarly preserved) immediately after defecation. For example, fluctuating temperature and humidity levels can influence bacterial enzymes that decompose steroid metabolites within exposed feces, thus increasing or decreasing the relative concentration of GCM measured in samples exposed to different environments (Khan et al. 2002; Terio et al. 2002; Millspaugh and Washburn 2004; Palme 2005; Shutt et al. 2012). Thus, effects of the environment on exposed samples can be difficult to separate from effects of the environment on physiological stress in the animal. Some studies can avoid this confounding factor by following individuals and collecting samples immediately after defecation (Creel et al. 1997) or by collecting samples deposited in snow (Creel et al. 2007). However, these collection protocols may not be possible for elusive species that are difficult to locate and monitor, or for territorial species that accumulate feces in latrine areas. In addition, for species that produce hard pellets (e.g., lagomorphs and some rodents), it can be difficult to estimate time since deposition.

**Figure 1:**
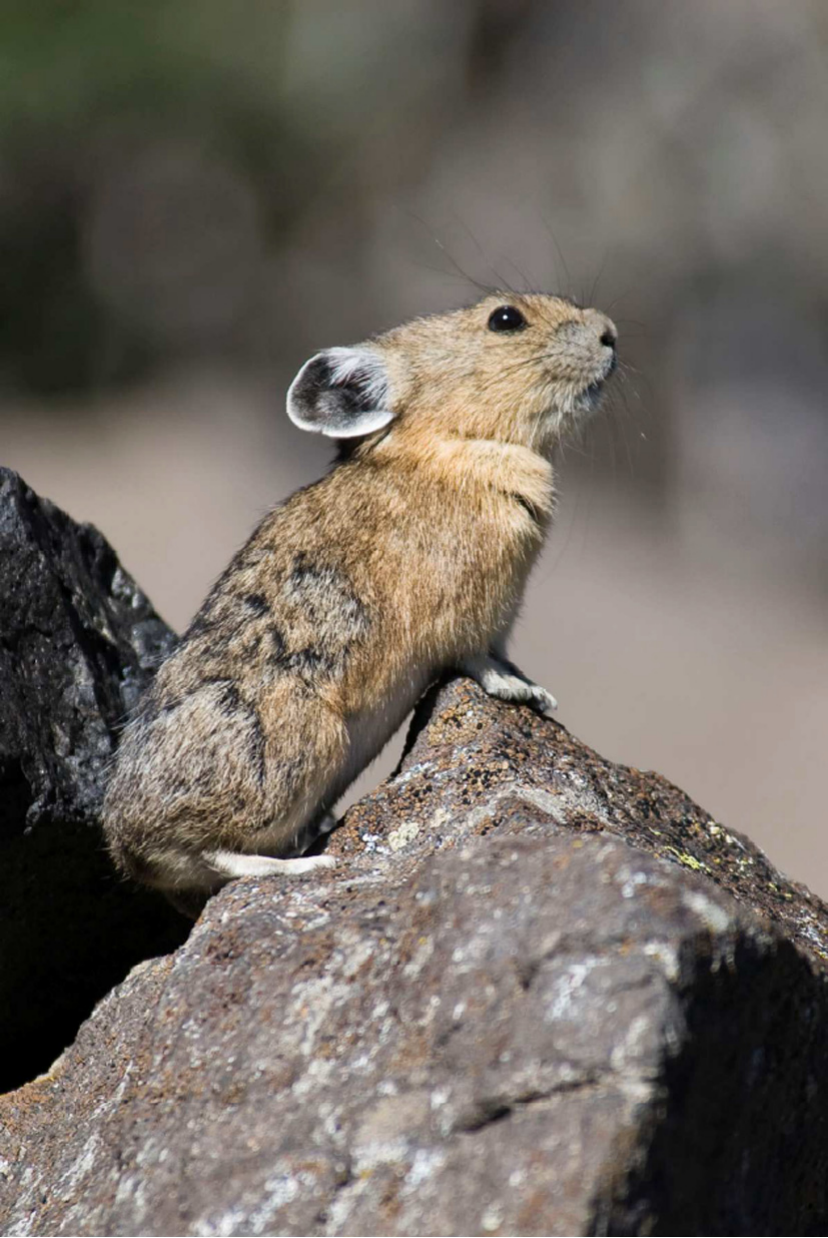
An American pika (*Ochotona princeps)*. Photo by Rebecca Barkley.

Here, we present results from the first multiregional assessment of physiological stress in an indicator species, the American pika (*Ochotona princeps*, Fig. 1). Pikas are a mammalian habitat specialist related to rabbits, which occur in rocky areas throughout the western United States. Due to their narrow thermal tolerance and recent population declines, they have been identified as an indicator species for identifying effects of anthropogenic climate change (McDonald and Brown 1992; Hafner 1993, 1994; Lawlor 1998; Beever et al. 2003; Smith et al. 2004; Grayson 2005). Our study highlights the first attempt to integrate physiological and climate data into population assessments for this species of conservation concern. Our first objective was to investigate the postdeposition influence of environmental conditions on GCM concentration in pika fecal samples. Because their latrines are easy to find, fecal sampling has become popular for monitoring pika occupancy and genetics (Millar & Westfall 2010, Nichols 2010; Jeffress and Garrett 2011; Castillo et al. 2014). However, fresh samples are difficult to obtain where microhabitat complexity makes it difficult to observe defecations. Our second objective was to evaluate factors that might predict GCM concentration across the pika's range. We used linear mixed‐effects models within an information‐theoretic framework to evaluate relative support for postdeposition and predeposition effects of climate and habitat characteristics on fecal GCM. Finally, we explored the scale at which postdeposition effects of the environment can outweigh predeposition effects on physiological stress in this species. Our study is unique in exploring pre‐ and postdeposition effects at multiple scales, and our findings have important implications for the use and interpretation of noninvasive stress measurement in populations of pikas and other wildlife.

## Materials and Methods

Pikas produce two types of feces: soft cecal feces, which are commonly re‐ingested, and hard fecal pellets, which are not re‐ingested (Smith and Weston 1990). Fecal pellets are commonly observed within pika territories, and pellets <1 week old can be distinguished from older pellets by color and consistency (Nichols 2010). In the current study, we focused on fecal pellets as a convenient means for noninvasive sampling.

### Influence of environment on GCM concentration

Fecal samples were collected from pikas captured in the Rocky Mountains within Boulder County, Colorado, USA. Pikas were live trapped, sampled during a 20‐min handling process under light anesthesia, and released at point of capture. Procedures were authorized by Colorado Parks and Wildlife (license no. TR2014) and approved by the University of Colorado‐Boulder Institutional Animal Care and Use Committee (protocol 1104.06).

A fecal sample consisting of approximately 20–40 pellets deposited naturally during the trapping and handling procedure was collected from each of 11 pikas. Only adult females were sampled, to control for known effects of age and sex on GCM concentration in pikas (Wilkening et al. 2013). Pellets were placed immediately on ice in the field, and transferred within 12 h to a −20°C freezer. Pellets were pooled across individuals and divided at random into three controls plus 24 samples slated for experimental exposure. Each “exposure sample” consisted of approximately 10 pellets inside a lidded, plastic, food‐storage container (9 cm × 9 cm × 12 cm) modified for aeration by mesh panel inserts (6 cm × 6 cm) on all six sides.

Timed exposures were conducted in triplicate at each of eight sites in two eco‐regions (Table 1). Exposure sites in the Rocky Mountains were Emerald Lake (EL) in Montana and three sites in Colorado: Niwot Ridge Long Term Ecological Research site (NWT), Brainard Lake Recreation Area (BRLA), and Rocky Mountain National Park (RMNP). Sites in the Oregon Cascades were Laurence Lake (LL) on Mount Hood and three sites in the Columbia River Gorge: Wyeth (WY), Herman Creek (HC), and Mosier Pass (MP). All sites contained numerous patches of typical pika habitat, characterized by large regions of broken rock (talus) interspersed with areas of vegetation. Sites represented a broad range of the habitats associated with pikas, from basalt lava flows located in river valleys to granite talus slopes above tree line (Fig. S1). Ranging 60–3625 m a.s.l., sites also included a variety of microclimatic conditions and vegetative communities.

**Table 1:** Fecal sample exposure sites (mean latitude, longitude, and elevation of exposure box positions

Eco‐region	Site name	Site abbreviation	Mean latitude	Mean longitude	Elevation range (m)
Rocky Mountains	Niwot Ridge LTER	NWT	40.06	105.60	3587–3625
	Brainard Lake Recreation Area	BLRA	40.07	105.59	3300–3326
	Rocky Mountain National Park	RMNP	40.40	105.67	3246–3313
	Emerald Lake	EL	45.41	110.93	2748–2846
Oregon Cascades	Laurance Lake	LL	45.43	121.67	928–933
	Wyeth	WY	45.69	121.80	160–172
	Herman Creek	HC	45.67	121.84	250–266
	Mosier Pass	MP	45.68	121.41	60–62

All sites except MP were occupied by pikas during this study. MP was approximately 25 km east of the distributional limit of pikas in the Columbia River Gorge (Simpson 2009). Habitat at MP was similar to occupied regions of the Gorge (WY, HC) in terms of average annual temperatures (ca. 10°C), but MP received considerably less precipitation (e.g., 40% less in 2012). We included MP in our exposure trials to extend the range of relative humidity and precipitation represented by study sites within this eco‐region.

At occupied sites, exposure samples were positioned next to fresh pika feces. At the unoccupied site (MP), samples were positioned in the talus under boulders, similar to a naturally occurring pika latrine. Pellets were kept on ice until placed into exposure containers on‐site. Containers were placed in natural voids under rocks to shield pellets from direct sunlight and rain, mimicking the typical environment of a natural latrine. All exposures were conducted during August 2012. After 2 weeks *in situ*, each exposure sample was stored on ice and transferred to the laboratory for analysis. Control samples were stored in a −20°C freezer during the entire exposure period, and GCM analysis was performed on all samples at the same time.

Extraction and analysis of GCMs was conducted using a commercially available Corticosterone Enzyme Immunoassay Kit (cat. no. K014‐H1; Arbor Assay Design, Inc., Ann Arbor, MI), as previously validated for pikas (Wilkening et al. 2013) Extracted samples were assayed in triplicate alongside a standard curve of seven known concentrations of corticosterone (5000–78,125 pg/mL). GCM concentrations for each sample were generated using a micro plate reader (BioTek Microplate Reader Synergy HT; 2005 Biotek Industries, Inc. Winooski, Vermont, USA) and Gen 5 1.11 Data Analysis software. Intra‐ and interassay coefficients of variation were 3–6 and 11–14%, respectively, and the cross‐reactivity of the antibody was 100% for corticosterone. Fecal GCM concentrations were expressed as ng GCM/g dry feces.

Monthly climate data with 800‐m resolution were obtained for each site from the PRISM climate group (www.prism.oregonstate.edu). From these data, maximum temperature, minimum temperature, and precipitation were calculated for the exposure month (August 2012) at each site.

Prior to analysis, data were checked for outliers, normal probability plots were examined, and the Shapiro–Wilk's statistic was calculated to test for normality. All data met the assumptions of normality. One‐way ANOVA was used to test for differences in GCM concentration among control samples and those exposed at different sites. Mean GCM concentration by site was regressed on maximum temperature, minimum temperature, and precipitation by site for the exposure month (August 2012) using linear mixed‐effects models to account for random site effects. Models were fitted using *lme4* (Bates et al. 2012). All statistical analyses were conducted using R 3.0.1 (R Core Team 2015), and significance was assessed at the *α *= 0.05 level.

### Multiregional assessment of GCM concentration

Fresh fecal pellets (Nichols 2010; Jeffress et al. 2013) were collected within ~1 week of deposition during summer 2010–2012 from 114 sites spanning seven states and almost the entire range of pika distribution in the western US. Collection sites ranged from high elevation talus slopes and boulder fields to low‐elevation lava beds.

The majority of samples were collected from western national parks by crews trained in a standardized collection and storage protocol designed to minimize contamination and maximize collection of fresh samples from unique individuals (Jeffress and Garrett 2011). Previous experiments demonstrated a negligible effect of storage on relative GCM concentration measured in samples collected using this protocol (Wilkening and Ray 2015). Extraction and analysis procedures followed those already described for the timed‐exposure study.

Given each sample's date and GPS location, PRISM climate data were summarized for each of three periods: the “postdeposition” period and two “predeposition” periods. We characterized climate during the postdeposition period by calculating PRISM estimates of maximum temperature, minimum temperature, and precipitation during the month in which the sample was collected. We characterized predeposition stressors based on pika population response to higher average summer temperatures, higher maximum summer temperatures, lower winter minimum temperatures, and lower amounts of winter precipitation (Beever et al. 2010, 2011; Erb et al. 2011; Wilkening et al. 2011; Jeffress et al. 2013). We defined the “summer stress” period as June to August of the year that the sample was collected. For this predeposition period, we calculated average temperature and average maximum temperature. We defined the “winter stress” period as October to May just prior to the summer in which the sample was collected. For this predeposition period, we calculated average minimum temperature and precipitation.

Vegetation data were collected at a subsample of randomly selected sites (*n* = 31) during the predeposition period following protocols in Jeffress and Garrett (2011). Each site was 12 m in radius and encompassed the fecal sampling location. Cover within each site was assigned to six categories: rock, bare ground, forb (flowering herbaceous plants), shrub (woody plants), grass (graminoids), and trees. Percent cover of each class was estimated using the midpoints of a modified Daubenmire scale (Daubenmire 1959 (Jeffress et al. 2013). Cover classes previously associated with pika dynamics were used as predictors, including forb cover, grass cover, and the ratio of grass to forb cover (Rodhouse et al. 2010, Wilkening et al. 2011, Jeffress et al. 2013, Erb et al. 2014).

In order to address repeated sampling from similar parks or eco‐regions (Buckley et al. 2003), we used linear mixed‐effects models to explain variation in GCM concentration. We evaluated the relative support for postdeposition and predeposition factors using eight candidate models. Models included postdeposition effects of climate on samples and/or predeposition effects on pikas (summer stress, winter stress, or available vegetation). Models incorporating vegetation were analyzed separately, because cover was estimated for only 31 of 114 sites. Models included pre‐ and/or postdeposition predictor variables as fixed effects and park or eco‐region as a random effect on model intercept and slope. Highly correlated (Spearman's *r* > 0.70) predictors were not used in the same model. GCM concentration was log‐transformed to correct for observed heteroskedasticity in residuals.

Models were developed using an information‐theoretic approach, fitted using *lme4*, and ranked by AIC_*c*_ (Burnham and Anderson 2002). For the subset of sites with full data (including vegetation cover estimates), predictor variables were ranked by Akaike weight, using the R package MuMIn (Barton 2014) to analyze all subsets of a global model including all 10 predictors and no interaction effects.

## Results

### Influence of environment on GCM concentration

**Figure 2:**
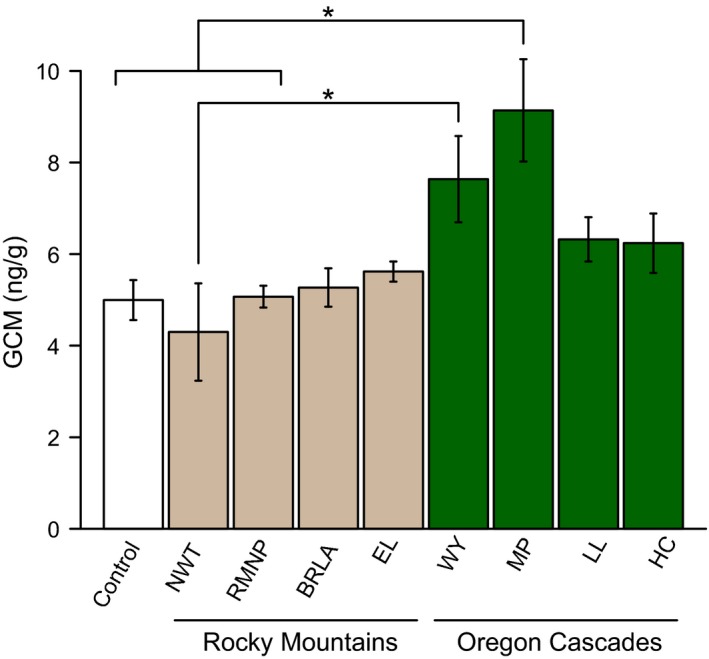
Mean glucocorticoid metabolite (GCM) concentration and standard error measured in control samples and samples exposed to local environmental conditions at eight sites across two eco‐regions. Site abbreviation is defined in Table 1. Asterisks identify sites where GCM concentration differed significantly as indicated by a post hoc Tukey test.

**Figure 3:**
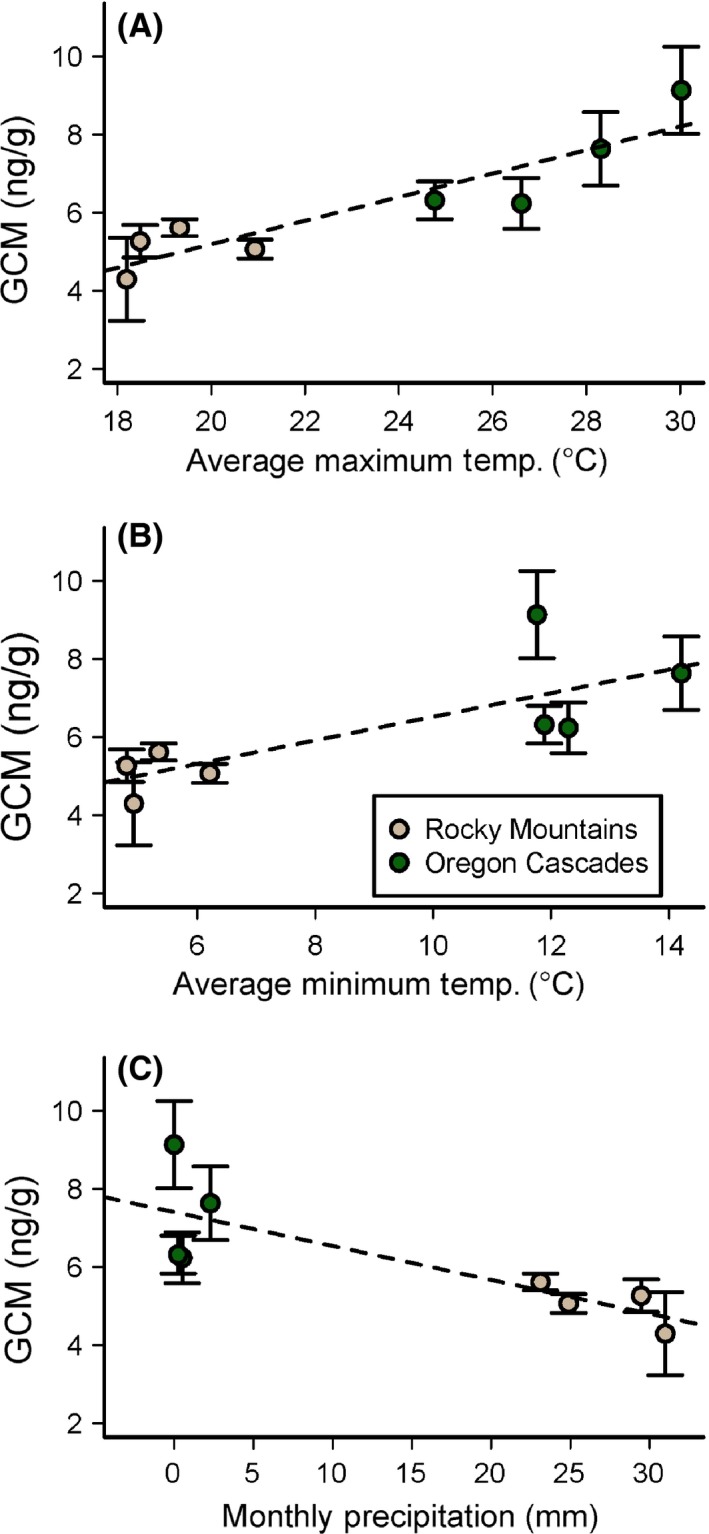
Mean and standard error of glucocorticoid metabolite (GCM) concentration measured in samples exposed at eight different sites in two eco‐regions, relative to (A) maximum temperature, (B) minimum temperature, and (C) precipitation during the month of exposure. Point color reflects eco‐region.

In our exposure experiment, GCM concentration varied significantly among control groups and samples exposed at different sites (*F*_(8, 18)_ = 4.39, *P* < 0.001; Fig. 2). A post hoc Tukey test showed significant differences in mean GCM concentration between WY and NWT (*P* = 0.04). GCM concentration in samples exposed at MP also differed significantly from those exposed at NWT, RMNP, and controls (all *P* ≤ 0.04). Mean GCM concentration in samples exposed at all other locations did not differ significantly from controls or between sites. Linear mixed‐effects models revealed significant relationships between GCM concentration and climate during the exposure month (Fig. 3), including positive effects of maximum temperature (*P* = 0.001) and minimum temperature (*P* < 0.001), and a negative effect of precipitation (*P* < 0.001).

### Multiregional assessment of GCM concentration

**Figure 4:**
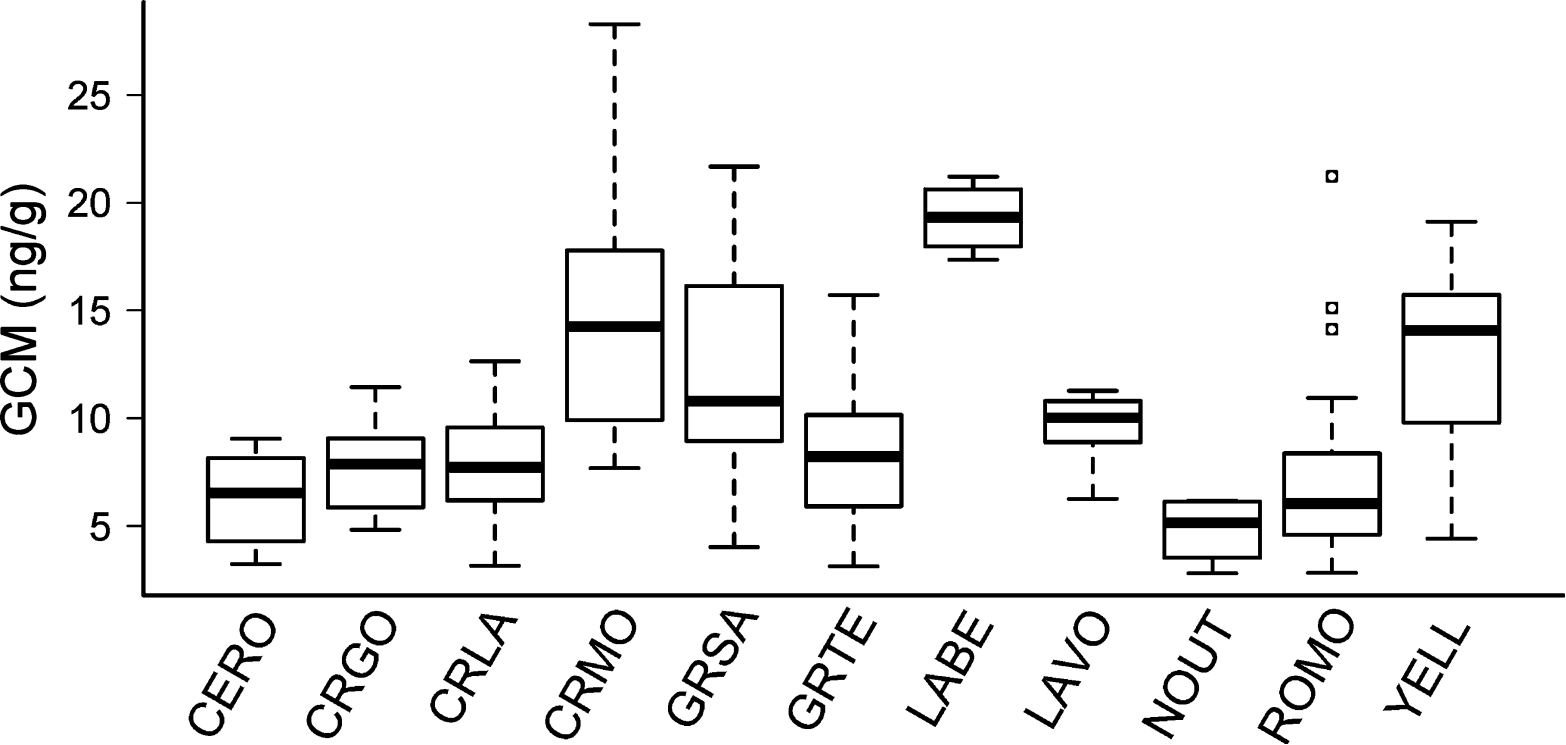
Glucocorticoid metabolite (GCM) concentration measured in samples collected from sites across the western US during 2010–2012. Boxes depict medians and 25% and 75% quartiles. Whiskers extend through the 95% interquartile range. Sites are grouped according to either national park or designated eco‐region. Abbreviations (in alphabetical order) are as follows: CERO = Central Rockies, CRGO = Columbia River Gorge, CRLA = Crater Lake National Park, CRMO = Craters of the Moon National Monument and Preserve, GRSA = Great Sand Dunes National Park and Preserve, GRTE = Grand Teton National Park, LABE = Lava Beds National Monument, LAVO = Lassen Volcanic National Park, NOUT = Northern Utah, ROMO = Rocky Mountain National Park, YELL = Yellowstone National Park.

Our multiregional comparison revealed considerable variation in GCM concentration measured in pika fecal samples from across the western US (Fig. 4). Among the 11 parks and other areas sampled, GCM was clearly highest in Lava Beds National Monument, California, and lowest in the Uinta Mountains, Utah.

Our *a priori* models of climate‐mediated GCM excluded interaction terms (which were nonsignificant) as well as collinear predictors. Thus, our models excluded one pairing among postdeposition effects (maximum and minimum temperature) and two pairings among predeposition effects: (1) average summer temperature and average maximum summer temperature, and (2) average summer temperature and average minimum winter temperature. There were no significant correlations among climate metrics representing post‐ and predeposition processes. Given these restrictions, the best climate model (Table 2) included only postdeposition effects (maximum temperature and precipitation during the sampling month). However, this model was rivaled by the null model, and one model with a predeposition effect (average summer temperature).

**Table 2:** Relative support for models of fecal GCM concentration (*n* = 114) including postdeposition effects of climate on the sample and predeposition effects of climate on physiological stress in pikas

Model	AIC_*c*_	ΔAIC_*c*_ a	*Β* b	*t* value	*P* value
PostDepMaxTemp^c^	664.03	–	0.27	2.14	0.04
PostDepPrecip			−0.01	−0.58	0.56
Null	664.09	0.06	–	–	–
PostDepMaxTemp	665.39	1.36	0.29	2.12	0.04
PostDepPrecip			−0.00	−0.40	0.69
PreDepAvgSumTemp^d^			−0.20	−0.88	0.38
PostDepMaxTemp	667.28	3.25	0.29	2.11	0.04
PostDepPrecip			−0.00	−0.40	0.69
PreDepMaxSumTemp			−0.21	−0.95	0.34
PreDepWinPrecip			−0.00	−0.12	0.90
PreDepMaxSumTemp	669.61	5.58	0.05	0.14	0.89
PreDepMinWinTemp			−0.16	−0.47	0.64
PreDepWinPrecip			0.00	0.33	0.74

a Models are ranked by ΔAIC_*c*_, the difference in AIC_*c*_ between the indicated model and the model with lowest AIC_*c*_.

b *Β * =  fitted coefficients for each predictor.

c Postdeposition predictors included maximum temperature (PostDepMaxTemp, °C) and precipitation (PostDepPrecip, mm) during the month in which each sample was collected.

d Predeposition predictors included average summer temperature (PreDepAvgSumTemp, °C), average maximum summer temperature (PreDepMaxSumTemp, °C), average minimum winter temperature (PreDepMinWinterTemp, °C) and total winter precipitation (PreDepWinPrecip, mm), all measured during the year preceding deposition of the sample.

Our *a priori* models of vegetation‐mediated GCM in 31 sites also excluded interaction terms (nonsignificant) and collinear pairings (grass:forb ratio and forb or grass cover). Vegetation related variables were significant predictors of GCM, and the null model had the lowest AIC score (Table 3).

**Table 3:** Relative support for models of fecal GCM concentration (*n *=* *31) including effects defined in Table 2 as well as effects of available forage on physiological stress in pikas. Postdeposition predictors are defined in Table 2. Pre‐deposition predictors include average maximum summer temperature (PreDepMaxSumTemp, °C), and total winter precipitation (PreDepWinPrecip, mm) measured during the year preceding deposition of the sample, and factors predictive of pika dynamics in previous studies such as cover of forbs (ForbCover), graminoids (GrassCover), and their ratio. Column headings are described in Table 2

Model	AIC_*c*_	ΔAIC_*c*_	*Β*	*t* value	*P* value
Null	189.09	–	–	–	–
ForbCover^a^	190.03	0.94	−0.03	−0.69	0.50
PostDepMaxTemp			1.02	2.14	0.04
PostDepPrecip			−0.60	−0.74	0.47
GrassForbRatio^b^	190.41	1.32	−0.08	−0.49	0.63
PreDepMaxSumTemp			0.60	1.71	0.10
PreDepWinPrecip			−0.00	−0.17	0.86
ForbCover	192.24	3.15	−0.01	−0.18	0.86
PreDepWinPrecip			−0.02	−1.28	0.21
ForbCover	193.04	3.95	0.01	0.20	0.84
GrassCover^c^			−0.01	−0.18	0.86

a Relative cover of forbs.

b Ratio of graminoid to forb relative cover values.

c Relative cover of graminoids.

**Figure 5:**
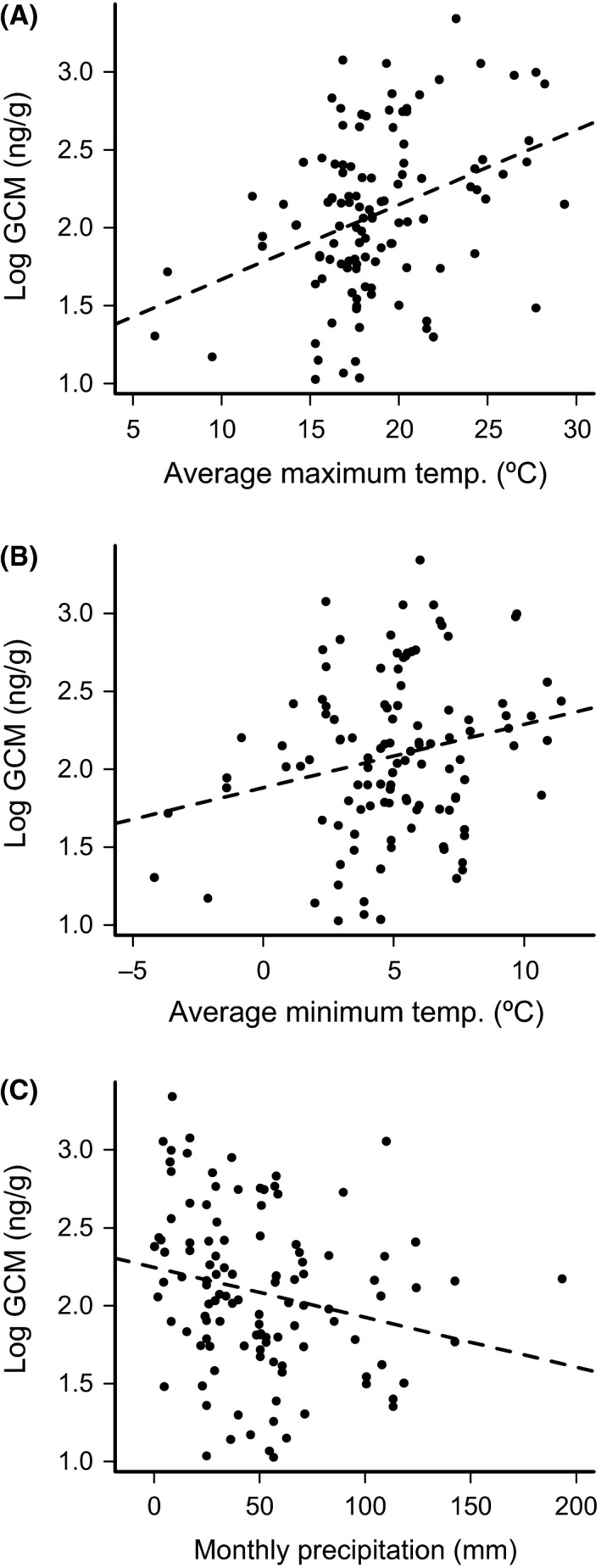
Glucocorticoid metabolite (GCM) concentration (log‐transformed) vs. postdeposition climate metrics: (A) maximum temperature, (B) minimum temperature, and (C) precipitation during the month of sample collection. Temperatures and precipitation were determined from monthly PRISM climate data.

Akaike weights indicated that postdeposition maximum temperature was the strongest predictor of fecal GCM in the 31‐site analysis of all 10 potential predictor variables. Overall, postdeposition and climatic predictors received higher support than predeposition and vegetation‐based predictors (Table 4). Postdeposition maximum temperature and minimum temperature were positively related to GCM concentration, while postdeposition precipitation was negatively related (Fig. 5).

**Table 4:** Relative support (Akaike weight) for post‐ and predeposition predictors of pika stress

Predictor	Akaike weight
PostDepMaxTemp	0.39
PostDepPrecip	0.37
PreDepMaxSumTemp	0.33
PreDepAvgSumTemp	0.31
PostDepMinTemp	0.23
PreDepMinWinTemp	0.22
GrassCover	0.21
GrassForbRatio	0.20
PreDepWinPrecip	0.18
ForbCover	0.18

## Discussion

Our results clearly indicate that postdeposition exposure to environmental factors influences GCM concentration when pika feces are not collected immediately after defecation and that noninvasive measurement of physiological stress in pikas across the western US may be confounded by the influence of localized environmental conditions. Our controlled exposure trials demonstrate that GCM concentration measured in pika fecal samples is sensitive to natural and ecologically relevant variation in temperature and precipitation. Metrics of physiological stress suggested by previous studies and measured prior to sample deposition failed to explain the substantial variation in fecal GCM concentration at the scale of the pika's range. Thus, we were not able to detect a range‐wide signal of climate‐induced physiological stress in pikas, given the confounding effect of local climate on fecal samples after deposition. Exposure to uncontrolled ambient conditions could be avoided if field samples were preserved immediately after defecation. This could be accomplished if pika latrines were located, cleaned of accumulated pellets, and monitored for defecation events, which precludes the opportunistic use of samples collected for occupancy or genetic studies. The pika's hard scat and the difficulty of observing pika defecation makes it challenging to collect fresh pika scat. Furthermore, pikas typically inhabit remote alpine areas which are difficult to access. As more species are targeted for noninvasive studies of physiological stress, similar challenges will be encountered more often.

Warmer postdeposition temperatures appear to increase measured GCM in pika fecal samples. This result was consistent between our experimental and observational studies, and has also been observed in laboratory experiments using fecal samples from other mammals. Experimental heating of fecal samples from white tailed deer (*Odocoileus virginianus*, Millspaugh et al. 2003) and cheetahs (*Acinonyx jubatus,* Terio et al. 2002) resulted in higher GCM concentration when compared to control samples. This pattern might be caused by increased microbial activity in response to warmer temperatures, which can increase decomposition of steroid hormones in the fecal sample (Terio et al. 2002; Millspaugh et al. 2003).

Postdeposition precipitation also negatively influenced GCM concentration in our analyses: the warmest and driest site (MP) had the highest measured GCM concentration. Other research related to the impacts of precipitation (or moisture) on GCM concentration has reported mixed effects. A study on white tailed deer (*O. virginianus*) feces found an increase in GCM concentration when samples were exposed to a simulated rainfall event. These increases were apparent even in samples exposed for short periods, and the additional moisture likely stimulated growth of the microbes that break down steroids (Washburn & Millspaugh 2002).

However, a study of grizzly bear (*Ursus arctos horribilis*) and American black bear (*Ursus americanus*) scat reported decreased GCM in response to precipitation (Stetz et al. 2013). This effect was attributed to steroid metabolite degradation in response to increased water on fecal samples, which could also account for our observed pattern.

While our results suggest consistent postdeposition environmental effects on GCMs across our broad study region, effects of local environmental conditions within the same eco‐region were not as pronounced. Half of our exposure sites were in the Rocky Mountains (NWT, BLRA, RMNO, EL), and the remaining sites were located in the Oregon Cascades (MP, LL, WY, HC). GCM concentration differed significantly only between samples experimentally exposed in different eco‐regions, and significant differences usually involved the one unoccupied site (MP; Fig. 2). We included this site because it is similar in macroclimate to other low‐elevation lava bed habitats occupied by pikas. However, low‐elevation sites occupied by pikas (e.g., WY, HC) may exhibit microclimatic conditions that differ significantly from the prevailing ambient conditions (Varner and Dearing 2014a). The fact that pikas were absent from MP suggests that this site may not support a microclimate suitable for pikas, and thus may not represent ecologically relevant exposure conditions for pika scat. Finally, among samples exposed within the Rocky Mountains, there were no significant differences between groups, nor were there differences between any group and controls frozen immediately after sampling Rocky Mountain pikas. Collectively, these results suggest that it may be possible to make valid comparisons among samples collected within the same eco‐region during the same season. Other studies have reliably correlated GCM concentration with habitat characteristics for a single population or several populations within the same region, where environmental variables are not likely to differ considerably (Balestri et al. 2014; Navarro‐Castilla et al. 2014; Davies et al. 2014; Rizo‐Aguilar et al. 2014). Results from our analysis spanning Colorado and Montana suggest that such comparisons may sometimes be justified even at very large scales.

Although there were considerable differences in the composition of vegetation across our study area, vegetation factors were not predictive of GCM concentration measured in pika scat. Even the grass:forb cover ratio, which varied from 0 to 30, did not predict residual GCM concentration in pikas. This result is surprising, given previous results suggesting that pikas thrive preferentially in locations with higher forb cover (Wilkening et al. 2011; Jeffress et al. 2013; Erb et al. 2014). Although pikas are generalist herbivores, they tend to consume plant species according to relative abundance and nutritional content (Huntley et al. 1986; Dearing 1996, 1997), and are increasingly selective when foraging in more extreme climates (Smith & Erb 2014). Our use of broad cover classes might weaken statistical relationships, but strong effects of forb and grass cover on pika occupancy have been detected using similar data (Jeffress et al. 2013).

The lack of a vegetation effect in our study may indicate that dietary fiber content does not differ considerably between forbs and grasses (i.e., food items; Varner and Dearing 2014b) in our samples. Dietary fiber may affect fecal mass and gut passage time, or it may influence gut microbial activities postdeposition that alter steroid metabolite structure (MacDonald et al. 1983; von der Ohe and Servheen 2002). Specifically, in herbivores, the level of fiber in the diet directly affects fecal bulk, and can therefore dilute GCM concentration in fecal samples (Goymann 2005). For example, in baboons (*Papio cynocephalus)*, an increase in dietary fiber led to a decrease in excreted progesterone metabolites (Wasser et al. 1993), and adding fiber to food decreased testosterone and corticosterone (GCM) in feces from European stonechats (*Saxicola rubicola;* Goymann 2005). However, testosterone and corticosterone (GCM) concentrations in fecal samples increased when the diet of red squirrels (*Tamiasciurus hudsonicus*) was supplemented with additional fiber (Dantzer et al. 2011). The effect of dietary fiber on GCM concentration requires further investigation.

Although proximate mechanisms are not well understood, variation in GCM concentration has been explained by diet in some species. The capercaillie (*Tetrao urogallus*), a European bird species, excreted higher concentrations of GCM when living in pine forests than when living in spruce forests, perhaps due to diet differences between the two habitat types (Thiel et al. 2011). Similarly, for Alaskan brown bears (*Ursus arctos horribilis*), a study examining multiple factors found the best predictor of fecal GCM concentration was diet type (von der Ohe et al. 2004). A more recent study on bears found that higher quality diets were associated with lower GCMs, identifying a nutritional influence on GCM concentration measured in fecal samples (Stetz et al. 2013). However, as we have shown, multiregional comparisons of GCM concentration between populations with access to different types or quality of food and different climatic conditions can be difficult to interpret, as changes in both dietary composition and climate may influence hormone metabolite concentration.

In conclusion, field studies relating noninvasive measurement of physiological stress to anthropogenic disturbance are increasing, and our results have several important implications for such studies. First, postdeposition environmental effects on exposed samples must be taken into consideration, especially for studies that have large geographical scope, or when samples cannot be preserved immediately after defecation. Second, comparisons within the same eco‐region may be reliable, particularly if samples are relatively fresh, sample size is large and there is knowledge of baseline values for regional populations. Finally, our multiregional comparison is an important step toward understanding how landscape scale patterns in climate and habitat might affect a species in decline across the western US. Previous pika studies have relied on the establishment of relationships between habitat characteristics and persistence or occupancy. However, the effects of climate change on populations start with individuals, and our use of a stress metric can be applied in future research to indicate habitat quality from the perspective of an individual animal. With further calibration to control for postdeposition effects, the baseline values that we measured for multiple eco‐regions should prove useful for population monitoring and for identifying environmental stressors before populations begin to decline. The prevalence of habitat loss and degradation underscores the importance of integrating such physiological measures into wildlife population assessments.

## Data Accessibility

All data have been uploaded online as Appendix S1.

## Conflict of Interest

None declared.

## Supporting information


**Figure S1.** Habitats used in timed‐exposure trials of pika fecal samples.

**Appendix S1.** Data used in all analyses.
