# Supplementary material for: When can we measure stress noninvasively? Postdeposition effects on a fecal stress metric confound a multiregional assessment
Source: Ecol Evol. 2016 Jan 9;6(2):502–13. doi: 10.1002/ece3.1857 (PMC4729247; doi:10.1002/ece3.1857)

**Supporting Information 1.** Habitats used in timed-exposure trials of pika fecal samples. Site abbreviations are listed in Table 1. In the Rocky Mountains: (A) NWT, (B) RMNP, (C) BRLA, (D) EL. In the Oregon Cascades: (E) WY, (F) MP, (G) LL, (H) HC.

This information is in support of an article submitted to Ecology and Evolution titled: “When can we measure stress non-invasively? Post-deposition effects on a fecal stress metric confound a multi-regional assessment ” by Jennifer Wilkening, Chris Ray and Johanna Varner.


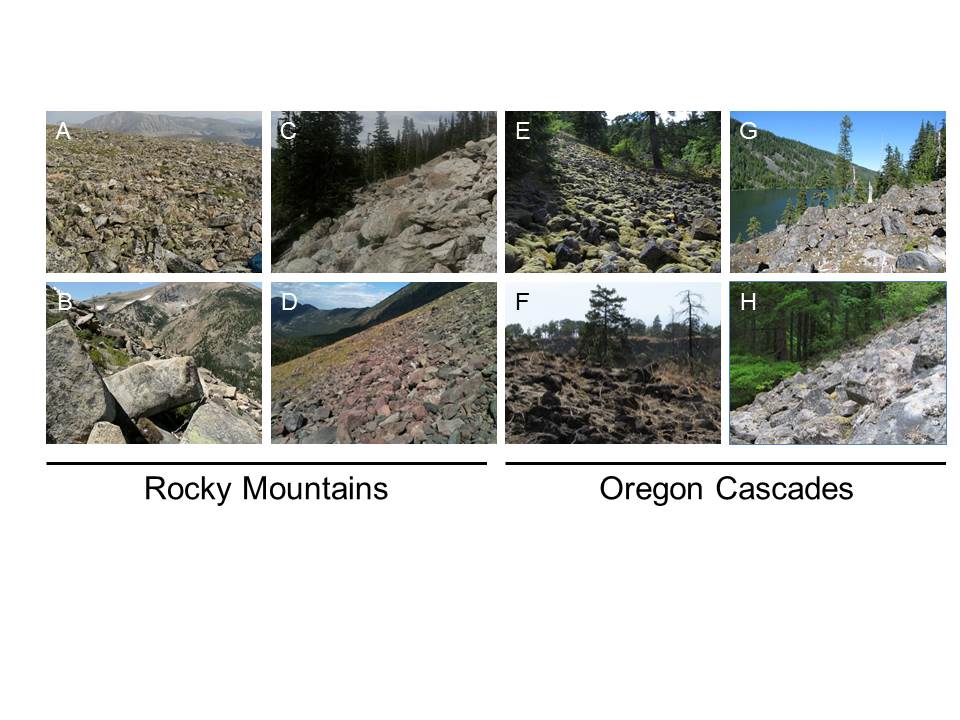

Supplement: Supplementary file 1 — Figure S1. Habitats used in timed‐exposure trials of pika fecal samples. [file ECE3-6-502-s001.docx]
