# Supplementary material for: When can we measure stress noninvasively? Postdeposition effects on a fecal stress metric confound a multiregional assessment
Source: Ecol Evol. 2016 Jan 9;6(2):502–13. doi: 10.1002/ece3.1857 (PMC4729247; doi:10.1002/ece3.1857)
Supplement: Supplementary file 2 — Appendix S1. Data used in all analyses. [file ECE3-6-502-s002.docx]

**Supporting Information 2.** Data used in all analyses.

This information is in support of an article submitted to Ecology and Evolution titled: “When can we measure stress non-invasively? Post-deposition effects on a fecal stress metric confound a multi-regional assessment” by Jennifer Wilkening, Chris Ray and Johanna Varner.
